# Supplementary material for: Testing Seefeldt’s Proficiency Barrier: A Longitudinal Study
Source: Int J Environ Res Public Health. 2022 Jun 11;19(12):7184. doi: 10.3390/ijerph19127184 (PMC9222670; doi:10.3390/ijerph19127184)
Supplement: Supplementary file 1 [file ijerph-19-07184-s001.zip › ijerph-1746242-Supplemantary file S1.pdf]

Supplementary file S1

Classes held in the motor intervention program

Class 1 – Warm-up; Catch all; Bounce the Ball in different directions; Bounce the ball freely around the court.<br>Class 2 – Warm-up; Relay race with and without bouncing the ball; Dribbling between cones and shooting into the basketball hoop (different types of ball, each student with a ball).<br>Class 3 – Warm-up; Catch all; Race with and without bouncing the ball; Dribbling between cones and shooting into the basketball hoop (different types of ball, each student with a ball).<br>Class 4 – Warm-up; Bouncing the ball in pairs; Catch one bouncing the ball.<br>Class 5 – Warm-up; perform the dribble and pass the ball to each other.<br>Class 6 – Warm-up; "Steal" your friend's ball; Catch one bouncing the ball just running in the court lines (each student with a ball).<br>Class 7 – Warm-up; Dribble between cones (each student with a ball): Game similar to Corfebol but you need bouncing the ball.<br>Class 8 – Warm-up; Dribble around the court (each student with a ball); Game similar to Corfebol but you need bouncing the ball.<br>Class 9 – Warm-up; Dribble around the court (each student with a ball); Mini basketball in pairs.<br>Class 10 – Warm-up; Mini basketball in quartets.
