# Supplementary material for: Testing Seefeldt’s Proficiency Barrier: A Longitudinal Study
Source: Int J Environ Res Public Health. 2022 Jun 11;19(12):7184. doi: 10.3390/ijerph19127184 (PMC9222670; doi:10.3390/ijerph19127184)
Supplement: Supplementary file 1 [file ijerph-19-07184-s001.zip › ijerph-1746242-Supplementary file S2.pdf]

Santos et al. - Proficiency Barrier Longitudinal

Organizing Data

I need just the first and second data collection, the sum of all six FMS skills and the dribble scores. Also, I will need the criteria for all bouncing and running (from the first data collection)

```
addpath('Data')
%Loading the data
aux=readtable('Base_Completa.xlsx');
%Creating the table that I will use for the analyses on the composite scores
soma=[aux.ID, aux.Sexo, aux.SOMA_CO_C1, aux.SOMA_CO_C2, aux.SOMA_SO_C1, aux.SOMA_SO_C2, ...
    aux.SOMA_SAT_C1, aux.SOMA_SAT_C2, aux.SOMA_QUI_C1, aux.SOMA_QUI_C2, aux.SOMA_RC_C1, ...
    aux.SOMA_RC_C2, aux.SOMA_CH_C1, aux.SOMA_CH_C2, aux.SOMA_DRIBLE_C1, ...
    aux.SOMA_DRIBLE_C2, aux.ERRO_DRI_C1,aux.ERRO_DRI_C2, aux.TEMPO_DR_C1,...
    aux.TEMPO_DRI_C2];
soma=array2table(soma,'VariableNames',{'id','sex','run1','run2','obs1','obs2','sat1',...
    'sat2','bou1','bou2','rec1','rec2','kic1', 'kic2','dri1','dri2','err1','err2',...
    'tdri1','tdri2'});
soma.dridelta=soma.dri2-soma.dri1;
soma.runbou1=soma.run1+soma.bou1;
soma.runbou2=soma.run2+soma.bou2;

soma=soma(~isnan(soma.run2) & ~isnan(soma.run1),:);

%Creating the table that I will use for the critical antecedents analysis
criterio=table2array(aux(:,[1,3,4,6:13,33:40,32,118]));
criterio=criterio(~isnan(criterio(:,end)) & ~isnan(criterio(:,end-1)),:);

doiscriterios=[criterio(:,4:7)+criterio(:,8:11),criterio(:,12:15)+criterio(:,16:19)];
doiscriterios=doiscriterios==2;
doiscriterios=double(doiscriterios);
```

Testing improvement in the different skills

I tested which skill individuals improved after a specific intervention on dribbling. I did all the tests using a bootstrapped t-test

```
addpath('C:\Users\mathe\Google Drive\MATLAB\Permutation')
```

```
Warning: Name is nonexistent or not a directory: C:\Users\mathe\Google Drive\MATLAB\Permutation
```

```
addpath('C:\Users\mathe\Google Drive\MATLAB\resampling')
```

```
Warning: Name is nonexistent or not a directory: C:\Users\mathe\Google Drive\MATLAB\resampling
```

```
comparison={'Skill','1st Wave','2nd Wave','t-value','DF','p-value'};
skills={'run','obstacle','one-leg jump','bounce','receive','kicking','driblling',...
    'errors in dribbling','time dribbling'};
for sk=1:9
    totest={table2array(soma(:,3+(sk-1)*2)),table2array(soma(:,2+sk*2))};
    comparison{sk+1,1}=skills{sk};
    var1 = table2array(soma(:,3+(sk-1)*2));
```

```
    var2 = table2array(soma(:,2+sk*2));
    eachIndMean = mean([var1,var2],2);
    grandMean = mean(eachIndMean);
    adjustmentFactor = grandMean - eachIndMean;
    comparison{sk+1,2} = [num2str(round(mean(var1),2)),' +- ',...
        num2str(round(std(var1),2))];
    comparison{sk+1,3} = [num2str(round(mean(var2),2)),' +- ',...
        num2str(round(std(var2),2))];
    [comparison{sk+1,4},comparison{sk+1,5},comparison{sk+1,6}]=statcond(totest, 'paired',...
        'on','mode','bootstrap','naccu',2000);
end
```

```
1 x 2, paired data, computing T values
Using paired t-test
Bootstraps (of 2000):...
1 x 2, paired data, computing T values
Using paired t-test
Bootstraps (of 2000):...
1 x 2, paired data, computing T values
Using paired t-test
Bootstraps (of 2000):...
1 x 2, paired data, computing T values
Using paired t-test
Bootstraps (of 2000):...
1 x 2, paired data, computing T values
Using paired t-test
Bootstraps (of 2000):...
1 x 2, paired data, computing T values
Using paired t-test
Bootstraps (of 2000):...
1 x 2, paired data, computing T values
Using paired t-test
Bootstraps (of 2000):...
1 x 2, paired data, computing T values
Using paired t-test
Bootstraps (of 2000):...
1 x 2, paired data, computing T values
Using paired t-test
Bootstraps (of 2000):...
```

```
disp(comparison)
```

```
  Columns 1 through 5
    {'Skill'            }    {'1st Wave'    }    {'2nd Wave'     }    {'t-value'}    {'DF'}
    {'run'              }    {'7.59 +- 0.85'}    {'7.87 +- 0.46' }    {[-3.6290]}    {[84]}
    {'obstacle'         }    {'4.12 +- 0.91'}    {'4.16 +- 0.91' }    {[-0.4062]}    {[84]}
    {'one-leg jump'     }    {'4.24 +- 1.36'}    {'4.21 +- 1.52' }    {[ 0.1428]}    {[84]}
    {'bounce'           }    {'6.12 +- 1.92'}    {'6.84 +- 1.49' }    {[-4.4799]}    {[84]}
    {'receive'          }    {'4.56 +- 1.28'}    {'4.55 +- 1.2'  }    {[ 0.0756]}    {[84]}
    {'kicking'          }    {'6.88 +- 1.33'}    {'7.14 +- 1.21' }    {[-1.7839]}    {[84]}
    {'driblling'        }    {'8.91 +- 3.91'}    {'10.13 +- 3.49'}    {[-4.6861]}    {[84]}
    {'errors in dribb…'}    {'1.66 +- 2.57'}    {'1.36 +- 2.48' }    {[ 1.2161]}    {[84]}
    {'time dribbling'   }    {'6.96 +- 2.23'}    {'6.32 +- 2.25' }    {[ 3.1162]}    {[84]}
  Column 6
    {'p-value'   }
    {[4.9975e-04]}
    {[    0.4943]}
    {[    0.7991]}
    {[4.9975e-04]}
    {[    0.9110]}
    {[    0.0085]}
    {[4.9975e-04]}
```

```
{[    0.0495]}
{[4.9975e-04]}
```

The first result is that, given the intervention, individual improve in running (p < .001), in bouncing (p < .001), kicking (p = .004), dribbling (p < .001), errors (p = .040) and time dribbling (p < .001). From these, only kicking was unexpected.

Testing for Cross-Sectional Proficiency Barrier

I'll do two things here. First, I'll check whether the previous equation can predict the barrier. It means that I'll restrict the previous equation in terms of the barrier location and will allow first and second constant to vary. Second, I'll fit the same equation to the second data collection data (need to check whether there are enough participants to fit).

```
%Fitting the linear model to compare against
[fitter_linear,stats_linear]=fit(soma.runbou2,soma.dri2,'poly1');
stats_linear
```

```
stats_linear = struct with fields:
          sse: 750.0253
      rsquare: 0.2673
          dfe: 83
   adjrsquare: 0.2584
         rmse: 3.0061
```

```

%Fitting with the constrained gamma and delta in the equation (gamma = 12.84, delta = 0.7586)
fo_constrained=fitoptions('Method','NonlinearLeastSquares','Lower',[0,0],'Upper',[16,16],...
    'StartPoint',[2,10]);
logist_constrained=fittype('c1+((c2-c1)/(1+exp(-(x-12.84)/0.7586)))','independent','x',...
    'options',fo_constrained);
[fitted_constrained,stats_constrained]=fit(soma.runbou2,soma.dri2,logist_constrained,...
    fo_constrained)
```

```
fitted_constrained =
     General model:
     fitted_constrained(x) = c1+((c2-c1)/(1+exp(-(x-12.84)/0.7586)))
     Coefficients (with 95% confidence bounds):
       c1 =       4.798  (2.798, 6.798)
       c2 =       11.36  (10.58, 12.14)
stats_constrained = struct with fields:
          sse: 742.7777
      rsquare: 0.2743
          dfe: 83
   adjrsquare: 0.2656
         rmse: 2.9915
```

```
fittedfunction=feval(fitted_constrained,[4:0.01:16]');
figure; plot(soma.runbou2,soma.dri2,'o','LineWidth',1.5)
hold on
plot([4:0.01:16],fittedfunction,'-.')
grid on
axis([4 16 0 16])

%Fitting without constraints
fo_unconstrained=fitoptions('Method','NonlinearLeastSquares','Lower',[0,0,10,0],...
```

```
    'Upper',[16,16,16,Inf],'StartPoint',[2,10,12,1]);
logist_unconstrained=fittype('c1+((c2-c1)/(1+exp(-(x-p0)/p1)))','independent','x',...
    'options',fo_unconstrained);
[fitted_unconstrained,stats_unconstrained]=fit(soma.runbou2,soma.dri2,logist_unconstrained,...
    fo_unconstrained)
```

```
fitted_unconstrained =
     General model:
     fitted_unconstrained(x) = c1+((c2-c1)/(1+exp(-(x-p0)/p1)))
     Coefficients (with 95% confidence bounds):
       c1 =      0.5265  (-12.52, 13.57)
       c2 =       11.76  (9.273, 14.25)
       p0 =       11.61  (8.267, 14.95)
       p1 =       1.397  (-0.7813, 3.575)
stats_unconstrained = struct with fields:
           sse: 732.7089
       rsquare: 0.2842
           dfe: 81
    adjrsquare: 0.2577
          rmse: 3.0076
```

```
fittedfunction=feval(fitted_unconstrained,[4:0.01:16]');
figure; plot(soma.runbou2,soma.dri2,'o','LineWidth',1.5)
hold on
plot([4:0.01:16],fittedfunction,'-.')
grid on
axis([4 16 0 16])

%Bootstrap to know the confidence interval of the boundaries
boots1=bootstrp(2000,@(x)[testlogist1(x),testlogist2(x)],[soma.runbou2,soma.dri2]);
```

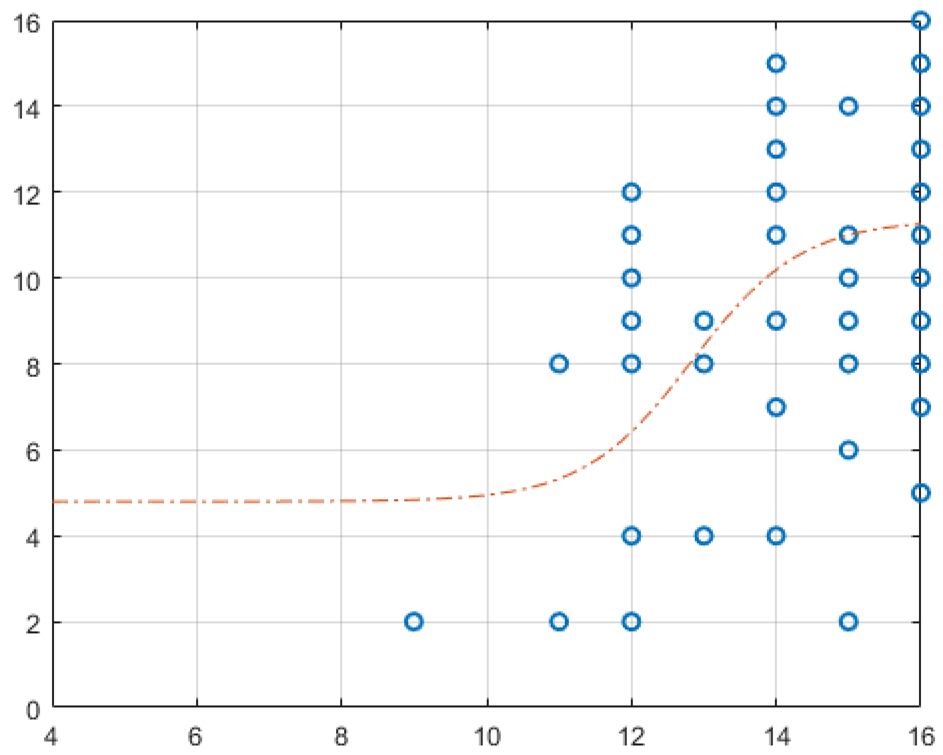

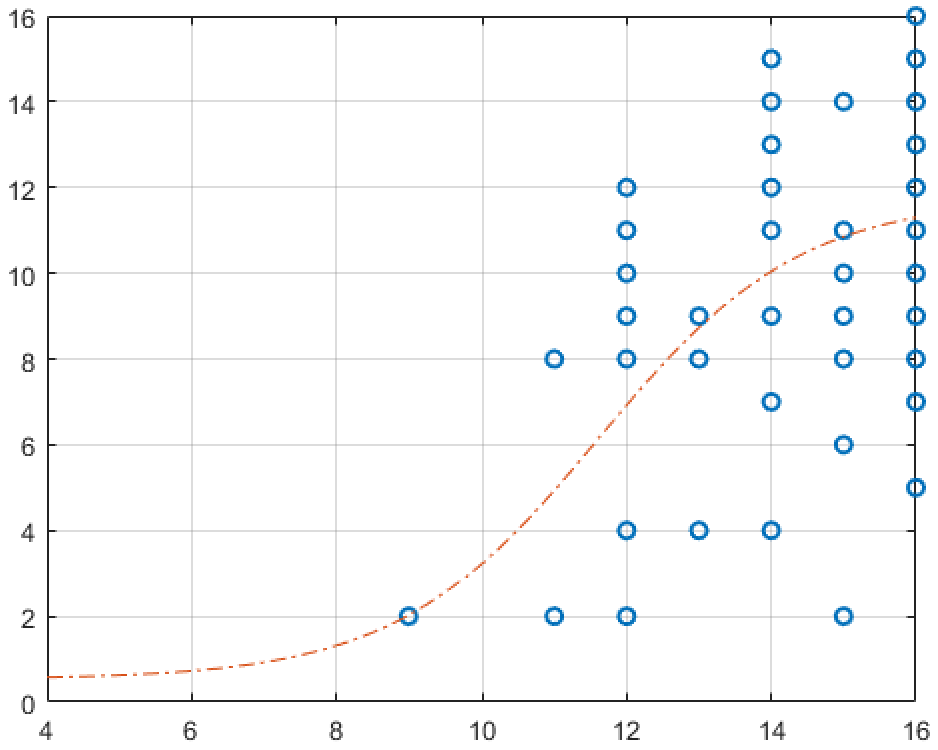


```
gamma=[mean(boots1(:,1)),prctile(boots1(:,1),2.5),prctile(boots1(:,1),97.5)]
```

```
gamma = 1×3
   12.2638   10.5574   15.5289
```

```
delta=[mean(boots1(:,2)),prctile(boots1(:,2),2.5),prctile(boots1(:,2),97.5)]
```

```
delta = 1×3
    1.1870    0.0284    3.4618
```

The first result is that the R-squared falls from the previous paper values to the current data set - which is not an issue. However, the sigmoidal curve explains more the data than the linear relation and the alpha and beta still encompass the values found in the previous paper. Clearly, the range of the data is increased.

When the unconstrained fitting is performed, the R-squared does not increase much and the previous study barrier value is maintained within the confidence interval.

When we perform the "joint" data set, the values again encompass the results of the previous study.

Testing the longitudinal barrier

For this part, I set the barrier values in terms of FMS and TMS and separate considering the first and second data collection. The idea is that only those with higher than the barrier at FMS 1 will be able to be above TMS 2. Even when considering that individuals improved in both FMS and TMS, the FMS2 and TMS 2 will still show a "barrier-like" association.

```
%Getting all children with higher than 12 on FMS
aboveFMS1=soma.runbou1>11;
```

```
aboveFMS2=soma.runbou2>11;
aboveTMS1=soma.dri1>9;
aboveTMS2=soma.dri2>9;
forSPSS=double([aboveFMS1,aboveFMS2,aboveTMS1,aboveTMS2,soma.dridelta]);

%Performing a bootstrap (fms1 x tms2, fms2 x tms2)
boots3=bootstrp(2000,@chisquared,[aboveFMS1,aboveTMS2]);
boots4=bootstrp(2000,@chisquared,[aboveFMS2,aboveTMS2]);
fms1tms2=[mean(boots3(:,1)),prctile(boots3(:,1),2.5),prctile(boots3(:,1),97.5)]
```

```
fms1tms2 = 1×3
   22.0151   10.8974   35.7487
```

```
fms2tms2=[nanmean(boots4(:,1)),prctile(boots4(:,1),2.5),prctile(boots4(:,1),97.5)]
```

```
fms2tms2 = 1×3
    5.8124    1.5179   13.0517
```

Thus, we found that the relation between FMS 1 and TMS 2 is significant (the critical value for p = .050 is 3.841; for p = .001 is 10.828) - there is an association between the proficiency barrier in FMS in the first data collection with being above 9 criteria in the TMS in the second data collection. This is not observed for FMS2 x TMS2 relation.

```
%Calculating the proficiency barrier
[crossfms1]=crosstab(aboveFMS1,aboveTMS2)
```

```
crossfms1 = 2×2
    11     0
    20    54
```

```
[crossfms2]=crosstab(aboveFMS2,aboveTMS2)
```

```
crossfms2 = 2×2
     3     0
    28    54
```

Note, however, that the barrier is maintained. That is, there is no instance of below FMS barrier being above TMS barrier.

Predicting the Proficiency Barrier with the Critical Antecedents

For this, I perform again the analysis from the first data collection to extract the critical antecedents factor. Then, I see whether it predicts the data as in the previous paper

```
%-----------------------------------------------------------------------------------------
% Categorical dimensions (we will use only the fms predicting the total on the dribbling
%-----------------------------------------------------------------------------------------
addpath(genpath('G:\My Drive\MATLAB\PA_polychoric'))

[factors_fms_cat,valfms_cat,vecfms_cat]=pa_rule_polychoric_missing(doiscriterios,99,2000,2);

%Creating the dimension of importance (only one got significance)
fmsfactor=doiscriterios*vecfms_cat(:,1);

%Fitting the antecedents of the first data collection to TMS in the second data collection
```

```
fo_unconstrained=fitoptions('Method','NonlinearLeastSquares','Lower',[0,0,1,0],'Upper',...
    [16,16,2.5,Inf],'StartPoint',[4,12,1.8,1]);
logist_unconstrained=fittype('c1+((c2-c1)/(1+exp(-(x-p0)/p1)))','independent','x',...
    'options',fo_unconstrained);
[fitted_antecedent,stats_antecedent]=fit(fmsfactor,soma.dri2,logist_unconstrained,...
    fo_unconstrained)
```

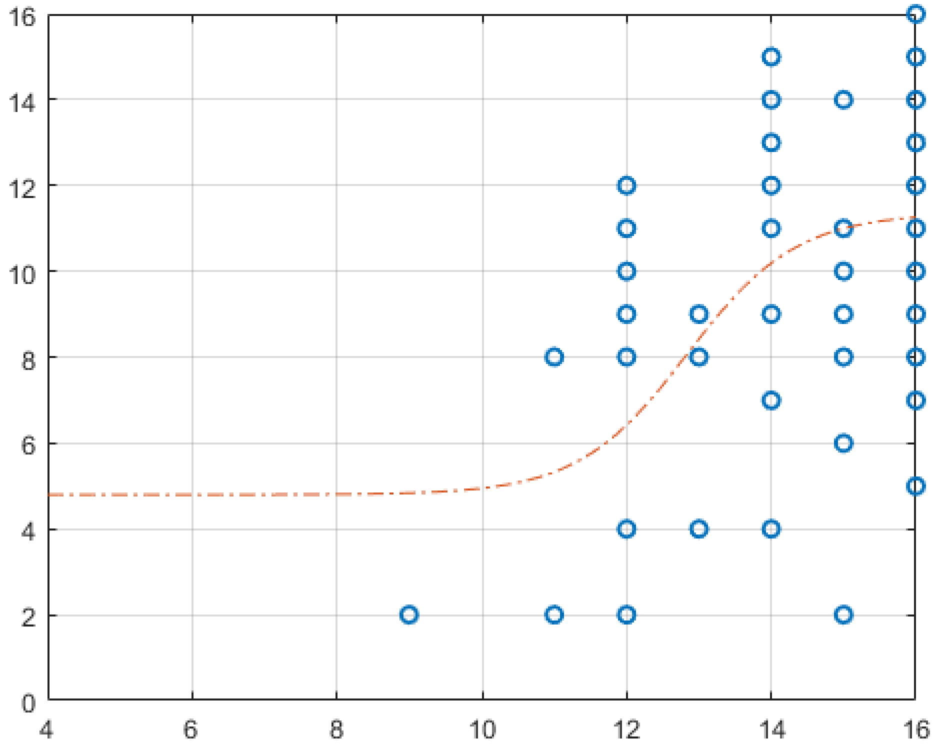

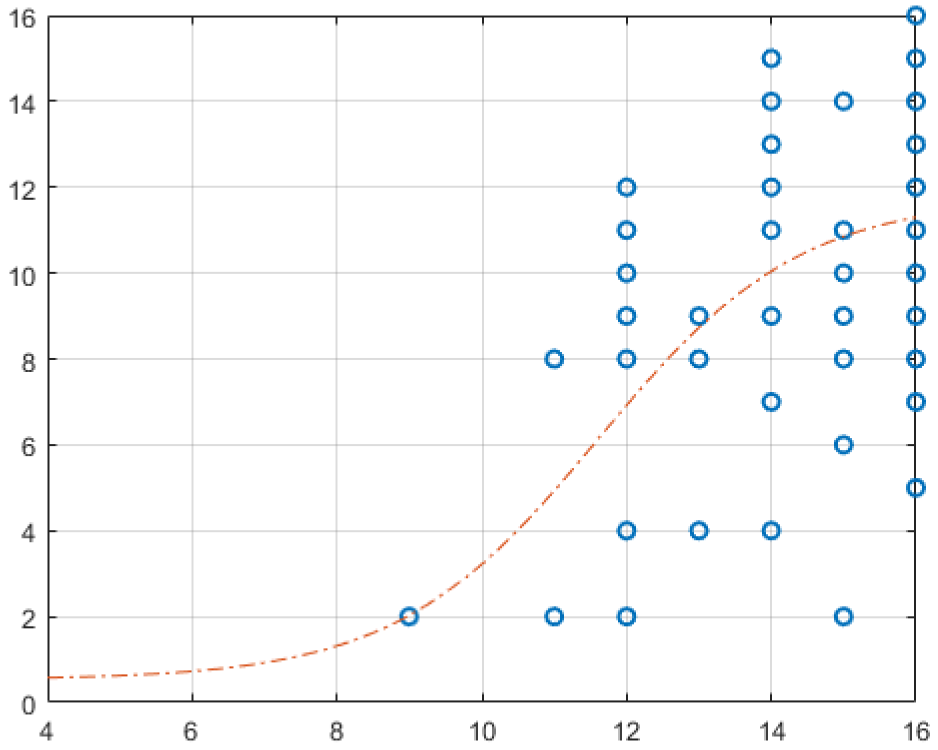


```
fitted_antecedent =
     General model:
     fitted_antecedent(x) = c1+((c2-c1)/(1+exp(-(x-p0)/p1)))
     Coefficients (with 95% confidence bounds):
       c1 =        5.91  (4.309, 7.512)
       c2 =       11.84  (10.95, 12.73)
       p0 =       2.051  (1.868, 2.235)
       p1 =       0.116  (-8.235e-05, 0.2321)
stats_antecedent = struct with fields:
           sse: 560.3759
      rsquare: 0.4525
          dfe: 81
   adjrsquare: 0.4323
         rmse: 2.6303
```

```
fittedfunction=feval(fitted_antecedent,[0.5:0.01:3]');
figure; plot(fmsfactor,soma.dri2,'o','LineWidth',1.5)
hold on
plot([0.5:0.01:3],fittedfunction,'-.')
grid on
axis([0.5 3 1.5 16.5])
```

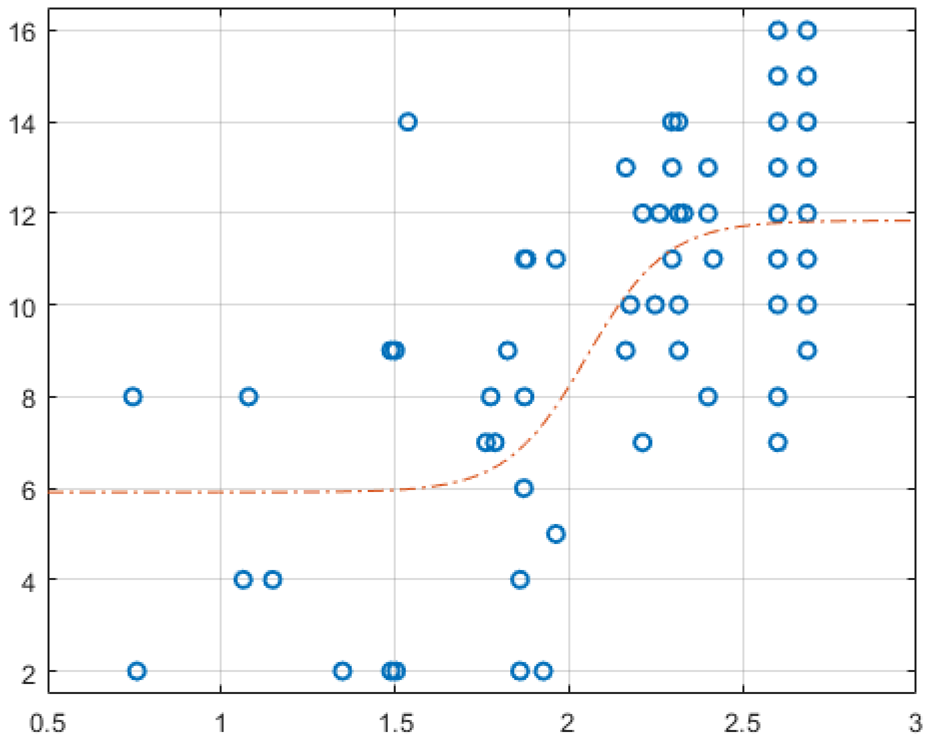

Thus, as supposed, only those with the critical antecedents at the first data collection showed high TMS at the second data collection.

Functions

These are only functions used in this script.

```
function [p0]=testlogist1(sample)
    fo_unconstrained=fitoptions('Method','NonlinearLeastSquares','Lower',[0,0,10,0],'Upper',...
        [16,16,16,Inf],...
        'StartPoint',[2,10,12,1]);
    logist_unconstrained=fittype('c1+((c2-c1)/(1+exp(-(x-p0)/p1)))','independent','x',...
        'options', fo_unconstrained);
    fitted_unconstrained=fit(sample(:,1),sample(:,2),logist_unconstrained,fo_unconstrained);
    p0=fitted_unconstrained.p0;
end

function [p1]=testlogist2(sample)
    fo_unconstrained=fitoptions('Method','NonlinearLeastSquares','Lower',[0,0,10,0],...
        'Upper',[16,16,16,Inf],...
        'StartPoint',[2,10,12,1]);
    logist_unconstrained=fittype('c1+((c2-c1)/(1+exp(-(x-p0)/p1)))','independent','x',...
        'options',fo_unconstrained);
    fitted_unconstrained=fit(sample(:,1),sample(:,2),logist_unconstrained,fo_unconstrained);
    p1=fitted_unconstrained.p1;
end
```

```
function [chi]=chisquared(sample)
    [~,chi,~]=crosstab(sample(:,1),sample(:,2));
end
```
